# Investigation of Inclusion Complex of Patchouli Alcohol with *β*-Cyclodextrin

**DOI:** 10.1371/journal.pone.0169578

**Published:** 2017-01-17

**Authors:** Fangfang Xu, Qiuxia Yang, Lilan Wu, Rui Qi, Yunshan Wu, Yucui Li, Lipeng Tang, De-an Guo, Bo Liu

**Affiliations:** 1 The Second Affiliated Hospital of Guangzhou University of Chinese Medicine, Guangdong Provincial Academy of Chinese Medical Sciences, Guangzhou, China; 2 Guangdong Pharmaceutical University, College of Traditional Chinese Medicine, Guangzhou, China; 3 College of Chinese Medicines, Guangzhou University of Chinese Medicine, Guangzhou, China; 4 Shanghai Institute of Materia Medica, Chinese Academy of Sciences, 501 Haike Road, Shanghai, China; University of Akron, UNITED STATES

## Abstract

The objective of this study was to improve the stability and water-solubility of patchouli alcohol by complexing with *β*-cyclodextrin (*β*-CD). The interactions between patchouli alcohol and *β*-CD were characterized by differential scanning calorimetry (DSC), Fourier transformation-infrared (FT-IR) spectroscopy, powder X-ray diffraction (PXRD), and Scanning electron microscope (SEM), respectively. According to molecular modeling method, the enthalpy formation of host-guest illustrated the predominant configuration and the lowest value *Δ*_*b*_*G*^*o*^ was -10.8174±1.9235 kcal/mol, suggesting the complex could reduce the energy of the system. The characterization analysis confirmed the formation of PA-CD inclusion complex, and the results indicated the advantage of the inclusion complex in stability and dissolution rates. These results identified PA-CD inclusion complex an effective way for the storage of PA, and better inclusion method still needed to be studied.

## 1 Introduction

**Fig 1:**
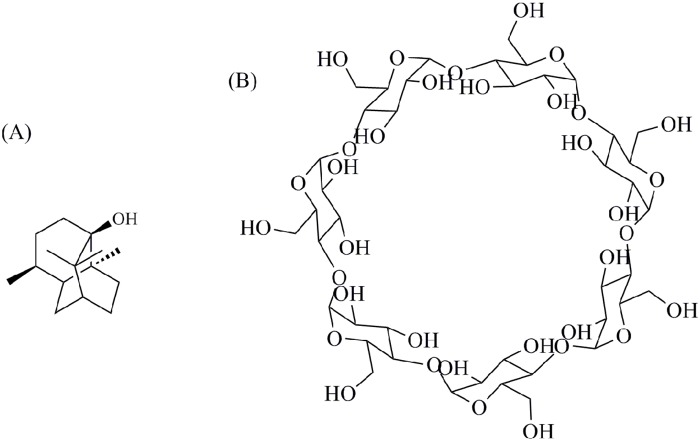
Chemical structures of (A) PA and (B) *β*-cyclodextrin.

Patchouli alcohol (PA, Fig 1) is a sesquiterpene with tricyclic structure, and has been extracted from the whole plant of traditional chinese medicine Guang-huo-xiang, which is also called *Pogostemon cablin* (Blanco) Benth. Patchouli alcohol is the nominal ingredient which standing for the typical aromatic odor and also used as the chemical reference for the quality control of *P*. *cablin* in Chinese Pharmacopoeia [1–2].

In traditional Chinese medicine, the *P*. *cablin* tasted hot, tepidity, owned to spleen, stomach and lung, usually used to treat cold, nausea and diarrhea [1]. PA has exhibited various pharmacological activities, such as protecting against the neurotoxicity of *β* amyloid peptide fragment 25–35 (A*β*_25–35_) [3], enhancing cognition in memory impairment mice induced by scopolamine [4], anti-inflammatory activities in RAW 264.7 cells and rats models [5–6], anti-influenza virus activities *in vitro* and *in vivo* [7–8]. However, Patchouli alcohol easily evaporates even at room temperature due to its volatile nature, which can cause the bioactivity decrease in the processing and storage. In addition, the development of Patchouli alcohol as medicine is greatly limited due to its low water solubility and poor bioavailability [9]. Therefore, searching for a safe and effective method for patchouli alcohol to enhance its stability and solubility became important.

Cyclodextrins (CDs) are a group of cyclic oligosaccharides consisting of 6–8 units of 1, 4-linked glucose units. The spaces of these macromolecules are expressed as circular table shape with different diameters [10]. The property of the inner cavity is hydrophobic, while the outer side is hydrophilic [11]. So the CDs performed as good host for water soluble and fat soluble compounds. The applications of CDs have been extensively investigated to improve the stability and solubility of poor water soluble compounds by formation of inclusion complexes [12–14].

Several works which focused on the reaction between cyclodextrins and volatile oils have been carried out [15–17]. The water-solubility of garlic oil was increased by forming inclusion complex with HP-*β*-CD [18].The stability of patchouli oil/*β*-CD complex was found higher than uncomplex oil [19]. The dissolution rate and oral bioavailability of PA solid dispersion with Eudragit have been improved through inhibiting reprecipitation in super saturated solution [20].

In this research, Patchouli alcohol and *β*-CD was prepared to form the inclusion complex with a saturated aqueous solution method, which was designed to improve the solubility and stability of PA [21]. Solubility phase analysis was performed to forecast the enhanced solubility of PA by *β*-CD. PA/CD inclusion was confirmed by differential scanning calorimetry (DSC), Fourier transform infrared spectroscopy (FT-IR), powder X-ray diffraction (PXRD), and Scanning electron microscope (SEM). Molecular modeling studies was carried out to obtain a three dimensional image of the most likely structure of the inclusion complex. Thermal stability, humidity stability, and photo-stability of PA-CD inclusion complex were compared with free PA to demonstrate the advantage of PA/CD inclusion complex.

## 2 Materials and Methods

### 2.1 Materials

Patchouli alcohol (PA, purity ≥ 99%) was gotten from Professor Su Ziren’s group (Guangzhou University of Chinese Medicine), *β*-Cyclodextrin (*β*-CD) was purchased from Boao Bio-Technology Co., Ltd. (Shanghai, China). Other reagents used in this study were analytical grade.

### 2.2 Preparation

The inclusion complex was prepared by saturated aqueous solution method [20]. PA (100 mg) was dispersed in water (100 mL) containing *β*-CD (1.0 g) and stirred for 4.0 h at 50°C. The resultant solution was filtered through a 0.45 μm syringe filter and then lyophilized (ALPHA 1–2 LD plus, CHRIST, Germany) for 36 h. The PA-CD inclusion complex was stored in a desiccator at 4°C until further use.

### 2.3 Solubility phase analysis

#### 2.3.1. Solubility phase method

Solubility phase analysis was manipulated in Thermo Max Q 4000 shaker (Thermo Scientific, USA) by the method reported by Higuchi and Connors [22]. The excessive amount of PA (10 mg) was added to volumetric flasks containing 5 mL various concentrations *β*-CD solutions (0, 0.9, 1.8, 3.6, 5.4, 7.2, and 9.1 mM) and then the solutions were ultrasonicated for 5 min. The flasks were then shaken continuously at 100 rpm in shaker at different temperatures (25 and 35°C) for 72 h and the suspensions were filtered through a 0.45 μm syringe filter. The amount of PA was analyzed by GC-MS spectrometry (Agilent 7890A/5975C, USA). Each experiment was performed in triplicate.

#### 2.3.2. GC-MS analysis

GC-MS analysis was carried out on an Agilent 7890A-5975C GC-MS system (Agilent, USA). The GC separation was conducted on a HP-5MS capillary column (30 m × 0.25mm, 0.25 μm). Nonsplit injection (0.5 μL) was conducted, and helium was used as carrier gas at the rate of 1.5 mL/min. The initial oven temperature was set at 90°C, and then programmed heating to 250°C by a gradient of 10°C/min (held for 2 min). The inlet temperature was 230°C. The mass spectrometer condition was: EI mode, the ionization energy: 70 eV, the ionization source temperature: 250°C, the scan range: 150–300 amu, and the scan rate: 0.25 s per scan.

### 2.4 Solubility test

Excess quantities of PA and its *β*-CD inclusion complex were dispersed in 25 mL of distilled water in sealed bottles to get a super-saturated solution. The bottles were shaken continuously for 24 h at ambient temperature until equilibrium was attained. Super-saturated solution was filtered through a 0.45 μm syringe filter and further diluted with methanol. The amount of PA was analyzed by GC-MS method mentioned in 2.3.2.

### 2.5 Characterization of the inclusion complexes

#### 2.5.1. Differential scanning calorimetry (*DSC*)

DSC method was used to check the formation of inclusion on a Netzsch STA449C thermal analyzer (Netzsch Corporation, Germany). The accurately weighted powdered samples of PA, *β*-CD, PA/CD IC (inclusion complex), and PA/CD PM (physical mixture) were laid in aluminum pans and heated from 20°C to 300°C at a scanning rate of 10°C min^−1^ under nitrogen gas flow (25 mL min^−1^).

#### 2.5.2. Fourier transform infrared spectroscopy (*FT-IR*)

The FT-IR spectra of PA, *β*-CD, PA/CD IC, and PA/CD PM were measured on a spectrometer (Perkin Elmer Spectrum 400, USA). The typical bands were recorded in the range of 4000 to 800 cm^−1^.

#### 2.5.3. Powder X-ray diffraction (*PXRD*)

The X-ray powder diffraction patterns of the powdered samples of PA, *β*-CD, PA/CD IC, and PA/CD PM were collected by copper radiation (40 kV, 20 mA), on a Ultima diffractometer (Empyrean, Nederland), in the range of 2 < 2θ < 60. The step size was 0.02° and the counting time was 2 s per step.

#### 2.5.4. Scanning electron microscope (*SEM*)

The scanning electron microscope was used to observe the appearance of surface morphologies of PA, *β*-CD, PA/CD IC, and PA/CD PM using a Jeol^®^JSM-5900. Powdered samples were fixed on a brass stub through double-sided tape and vacuum-coated gold.

### 2.6 Molecular modeling

In order to investigate and confirm the inclusion behavior of guest patchouli alcohol (PA) into host (*β*-CD), autodock 4.2.3 was used to simulate the supermolecular structure of the inclusion complex with regular genetic algorithm method [23].The docked conformation which had the least binding energy was selected to analyze the mode of binding. The interaction of host-guest were developed by PyMol molecular viewer [24].

Amber 11 Molecular Mechanics/Generalized Born Surface Area (MM/GBSA) program was used to calculate the binding delta Gibbs free energy. The docking result was also optimized by molecular simulation using amber 11 program [25]. The optimization ran for 2 ns. The binding energy (*Δ*_*b*_*G*^*o*^) was calculated according to Eq (1):

$$\Delta_{b}G^{o}=E_{complex}-E_{host}-E_{guest}$$ (1)

The calculated energy of PA, *β*-CD and the inclusion complex molecules were E_host_, E_guest_, and E_complex_ (kcal/mol), respectively.

### 2.7 Stability studies

The effect of inclusion complex on the thermal stability, humidity stability, and photostability of free and complexed PA with *β*-CD were examined on a thermo statically controlled stability chamber (SHH-SDT, Yongsheng Gallenkamp, China) under the following stress conditions. The thermal stability of PA powder samples were tested at 40°C, the humidity stability of PA powder samples were examined at 25°C / 70% relative humidity (RH), and the photostability tests were conducted under 4500 lx at 25°C [14]. During the testing period of 10 days, all experiments were carried out in triplicate. The samples were dissolved and diluted into appropriate concentrations by methanol, and the content of PA was examined by the GC-MS method described above in 2.3.2.

## 3 Results and Discussion

### 3.1 Solubility phase study

**Fig 2:**
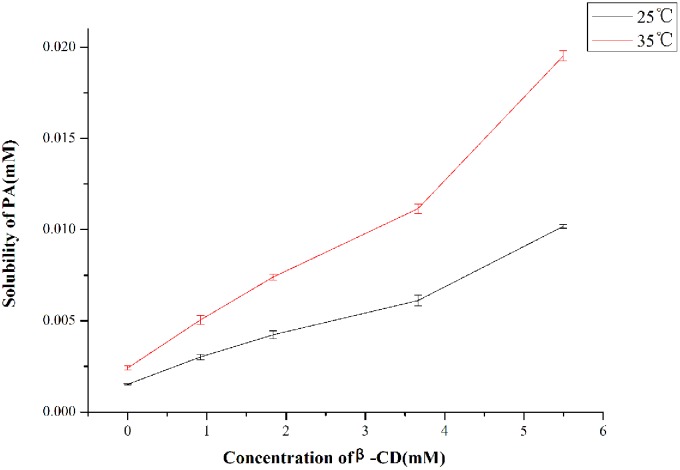
Phase solubility diagram of PA and *β*-CD at 25, 35°C (n = 3).

Solubility phase analysis was performed to detect the solubilizing ability of *β*-CD to PA and the inclusion stability constant of PA/CD complex inclusion. Fig 2 shows the solubility phase diagrams of PA-*β*-CD inclusion in two different temperatures (25, 35°C). Both diagrams can be viewed as A_L_-type, indicating that the solubility of PA was increased linearly with the raise of the CD concentration, and the stoichiometric ratio was 1:1.

The inclusion stability constant *K*_1:1_was defined by the Higuchi eq (2), where *S*_0_ represented the equilibrium solubility of PA in water and slope was gained from the solubility phase diagram [22, 26]. The *K*_1:1_ at 25, 35°C was calculated as 932.0 and 997.6 M^-1^, respectively:

$$K_{1:1}=\frac{\text{slope}}{S_{0}(1-\text{slope})}$$ (2)

### 3.2 Characterization

When the guest molecules were included into the cyclodextrin cavities, their physical characteristics, such as melting, boiling and sublimation points will change [27].

#### 3.2.1. DSC analysis

**Fig 3:**
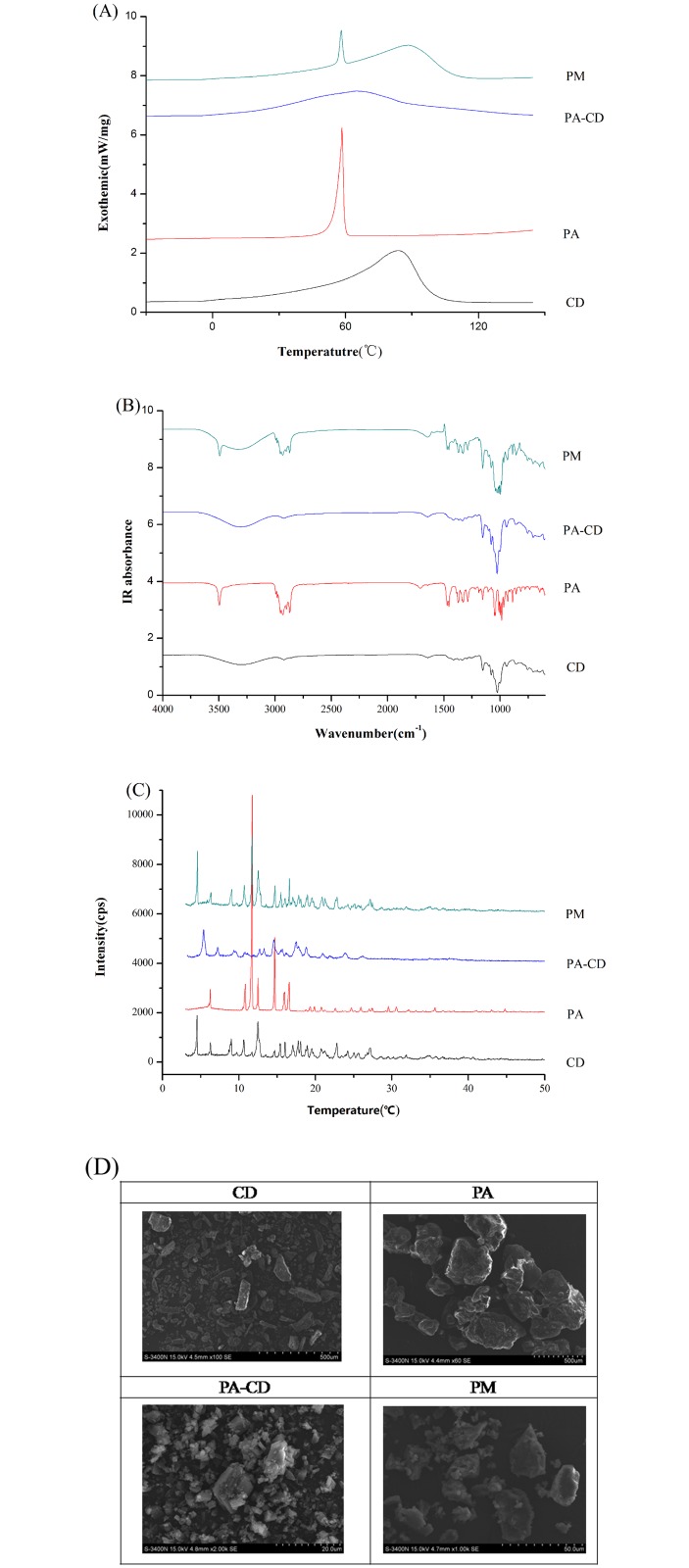
Characterization of PA, *β*-CD, PA/CD CI, and PA/CD PM. (A) DSC thermograms. (B) IR spectra. (C) 6 PXRD patterns. (D) SEM spectra.

The DSC thermograms of PA, *β*-CD, PA/CD IC, and physical PA/CD PM were shown in Fig 3A. The thermogram of PA showed narrow sharp peak at 58.4°C, while which of *β*-CD exhibited a broad blunt peak ranging from 60 to 100°C, and the peak emerged at 83.8°C, indicating the melting points of PA and *β*-CD. For the PA/CD PM, the endothermic peaks of PA and the *β*-CD were both observed, indicating the physical property of mixture sample was similar to the two components, and the PM sample was still the simple mixture of PA and the *β*-CD. As concerned to the complex inclusion, the complete disappearance of sharp PA peak at 58.4°C and the broad *β*-CD peak at 83.8°C, and the shift of peaks at 65.0°C indicating that some interactions were established between PA and the *β*-CD. In a word, the DSC results suggested that PA-*β*-CD complex inclusion was successfully formed.

#### 3.2.2. FT-IR analysis

FT-IR spectra of *β*-CD, PA, PA/CD IC, and physical PA/CD PM were illustrated in Fig 3B. The spectrum of PA showed a distinct peak in the 3500 cm^−1^, indicating the presence of OH group. The bands in 3000 to 2850 cm^−1^ regions were assigned to saturated C-H stretching vibration. The bands at 1460, 1380 cm^−1^, and 1470 cm^−1^ were represented the bending vibration (CH_3_, CH_2_) from the ring of PA molecule. In the spectrum of *β*-CD, the broad peak at 3271 cm^−1^ could be assigned to the multi-hydrogen bonds. The spectrum of physical mixture was the simple combination of PA and *β*-CD, many characteristic peaks of PA at 3500, 1460, 1380, and 1470 cm^−1^and the classic broad *β*-CD peak at 3270 cm^−1^ were easily found, suggesting that PA and *β*-CD were independent existence without any interaction between each other. For the complex inclusion, the characteristic IR peaks of PA were almost completely vanished, indicating the new structure was formed and the guest molecule PA was entirely embedded into the internal space of *β*-CD.

#### 3.2.3. PXRD analysis

Fig 3C showed the PXRD patterns of *β*-CD, PA, PA-CD CI, and PA-CD PM. The existence of intense and acute peaks in the PXRD of PA (2θ = 10.8, 11.7, and 14.7°) and *β*-CD (2θ = 9.0, 12.5, 22.8 and 27.2°) indicated their crystalline form. In physical mixture, the classic peaks of PA and *β-*CD were observed indicating that the crystalline structures of the PA and *β-*CD were still kept without any change. The spectrogram of PA-CD inclusion complex showed distinctive broad bands, indicating new complex inclusions were successfully formed.

#### 3.2.4. SEM analysis

The differences between particles were observed by electronic microscopy shown in Fig 3D. *β*-CD particles presented a rod shaped form while PA presenting a cubic shape. For PM, which is a mixture of raw materials, as described for PA and *β*-CD, particles' shapes and sizes didn't change after mixing. PA/CD complex inclusion particles presented the cube-shaped form, the size was smaller and different from the raw materials' morphology. These observations confirm that a different entity (inclusion complex) was successfully formed.

### 3.3 Molecular modeling studies

**Fig 4:**
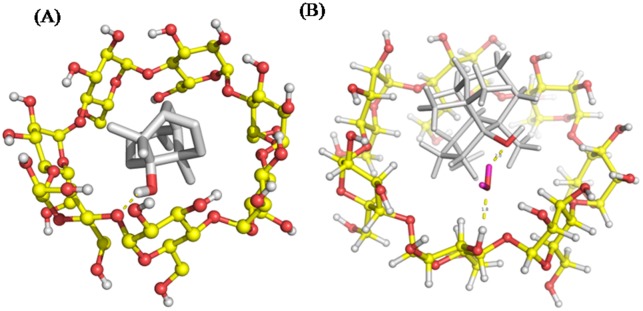
Lowest energy PA-*β*-CD docked complex. (A) Stick model. (B) The optimized model. Yellow stick represents *β*-CD and grey small molecule represents PA.

The molecular docking study was carried out to illuminate more about the geometry configuration of the PA-CD inclusion. The preferential relative orientation for the complex was shown in Fig 4A. The binding energy (*Δ*_*b*_*G*^*o*^) was calculated below (Table 1). Moreover, the binding mode was also optimized (Fig 4B).

**Table 1:** The interaction energy between PA and *β*-CD calculated with amber 11 program.

*E*_*complex*_ (kcal/mol)	*E*_*host*_(kcal/mol)	*E*_*guest*_(kcal/mol)	*Δ*_*b*_*G*^*o*^(kcal/mol)
-781.2432±9.4962	-743.7955±8.9522	-26.6304±1.8566	-10.8174±1.9235

Host:*β*-CD; Guest:PA

The binding energy (*Δ*_*b*_*G*^*o*^) of complex and the isolated molecule (PA and *β*-CD) suggested that stability of complex. The lower values for complexation energy meant the more stable complex. *Δ*_*b*_*G*^*o*^ of the complex was calculated with the minimum energy mode according to Eq (1) and the data of *Δ*_*b*_*G*^*o*^ were -10.8174±1.9235 kcal/mol.

From the 3D structure with the minimum energy mode presented in Fig 4, the free guest molecule PA was found to be completely entrapped into the *β*-CD cavity as the length of *β*-CD cavity were greater than that of PA and formed a cylindrical structure.

In the molecular dynamics simulation, one water molecule was added to the inclusion system by the TIP3P model. The hydrogen bonds were formed between the hydroxyl group in PA molecule and the nearby water molecule, and between the water molecule and the cyclodextrin molecule. Therefore, the water-bridged hydrogen bond was formed when the water molecule used as mediation. And the measured distance of hydrogen bonds were 2.0 and 1.8 Å respectively.

### 3.4 Stability

**Fig 5:**
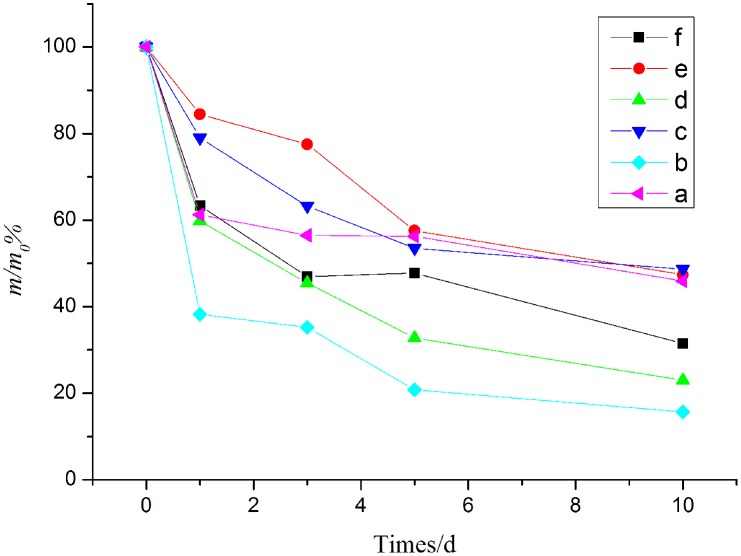
Degradation profiles of PA-CD inclusion complex (a, c, e) and PA (b, d, f). Thermal stability (a, b), humidity stability (c,d), and photostability (e,f).

The amount of changes of PA, PA/*β*-CD was tracked by GC-MS to evaluate the stability. Fig 5 illustrates the trends of change in the relative amount *m*/*m*_0_ of PA and PA/*β*-CD respectively. The relative amount of free PA showed a quickly degradation behavior under the 40°C, 70% relative humidity, and 4500 lx (Fig 5b, 5d and 5f), suggesting the poor stability of PA. However, when PA was included with *β*-CD, the degradation rate of PA was much slower in the overall process as shown in Fig 5a, 5c and 5e). This indicated that the stability of PA in high temperature, high humidity, and strong light were improved through the complex with *β*-CD.

### 3.5 Solubilization test

After complexation with *β*-CD, the water solubility of PA was slightly enhanced and increased from 12.66 μg/mL (*RSD* = 5.28%) to 17.49 μg/mL (*RSD* = 1.39%), which indicated the formation of a relative better water-soluble inclusion complex.

## 4 Conclusion

In this study, the inclusion complexes formed between PA with *β*-CD were studied by phase-solubility analysis, FT-IR, DSC, PXRD, and SEM technologies. The molecular modeling suggested that the complex of 1:1 host—guest had the lowest *Δ*_*b*_*G*^*o*^ value, the PA molecular was totally entrapped into the *β*-CD cavity. The stability of PA was significantly enhanced by inclusion. The water solubility of PA was slightly enhanced by inclusion with *β*-CD and better inclusion methods are still needed to be studied. Given the limitation of applications of free PA, the convenient preparation and the advantage of the PA/CD complex, this inclusion method should be deemed a prospective strategy for further utilization of PA.
